# Supplementary material for: Tree‐ensemble analysis assesses presence of multifurcations in single cell data
Source: Mol Syst Biol. 2019 Mar 27;15(3):e8552. doi: 10.15252/msb.20188552 (PMC6437440; doi:10.15252/msb.20188552)
Supplement: Supplementary file 1 — Appendix [file MSB-15-e8552-s001.pdf]

**Appendix Figures** 2

Appendix Figure S1: Comparison of methods on synthetic data containing no branch points 2

Appendix Figure S2: TreeTop applied to T cell thymic maturation data, with full markers 4

Appendix Figure S3: TreeTop applied to B cell maturation data, with full markers 6

Appendix Figure S4: Wishbone applied to T cell thymic maturation data, with full markers 8

Appendix Figure S5: Wishbone applied to B cell maturation data, with full markers 10

Appendix Figure S6: Monocle applied to T cell thymic maturation data, with full markers 11

Appendix Figure S7: Monocle applied to B cell maturation data, with full markers 13

Appendix Figure S8: TreeTop applied to healthy bone marrow data, with full markers 14

Appendix Figure S9: Gating strategy for healthy bone marrow data 16

Appendix Figure S10: TreeTop applied to synthetic branching data, with full markers 18

Appendix Figure S11: Comparison of molecular species abundance distributions of synthetic branching data with mass cytometry data 20

Appendix Figure S12: Distribution of annotated celltypes in TreeTop applied to healthy human bone marrow single cell RNA-seq data from Paul et al 21

Appendix Figure S13: Distribution of annotated celltypes in TreeTop applied to healthy human bone marrow single cell RNA-seq data from Velten et al 22

Appendix Figure S14: Robustness of TreeTop to number of reference nodes 23

Appendix Figure S15: Distributions of raw branching scores on permuted data 24

Appendix Figure S16: Effect of dimensionality and topology of synthetic data on raw branching scores 25

Appendix Figure S17: Effect of input parameters on reference score distributions 26

**Appendix Tables** 28

Appendix Table S1: Details of synthetic datasets 28

Appendix Table S2: Generation of hierarchically branching synthetic data 28

Appendix Table S3: TreeTop parameters 29

Appendix Table S4: Wishbone parameters 30

Appendix Table S5: Timings for comparison methods 30

# Appendix Figures

## Appendix Figure S1: Comparison of methods on synthetic data containing no branch points

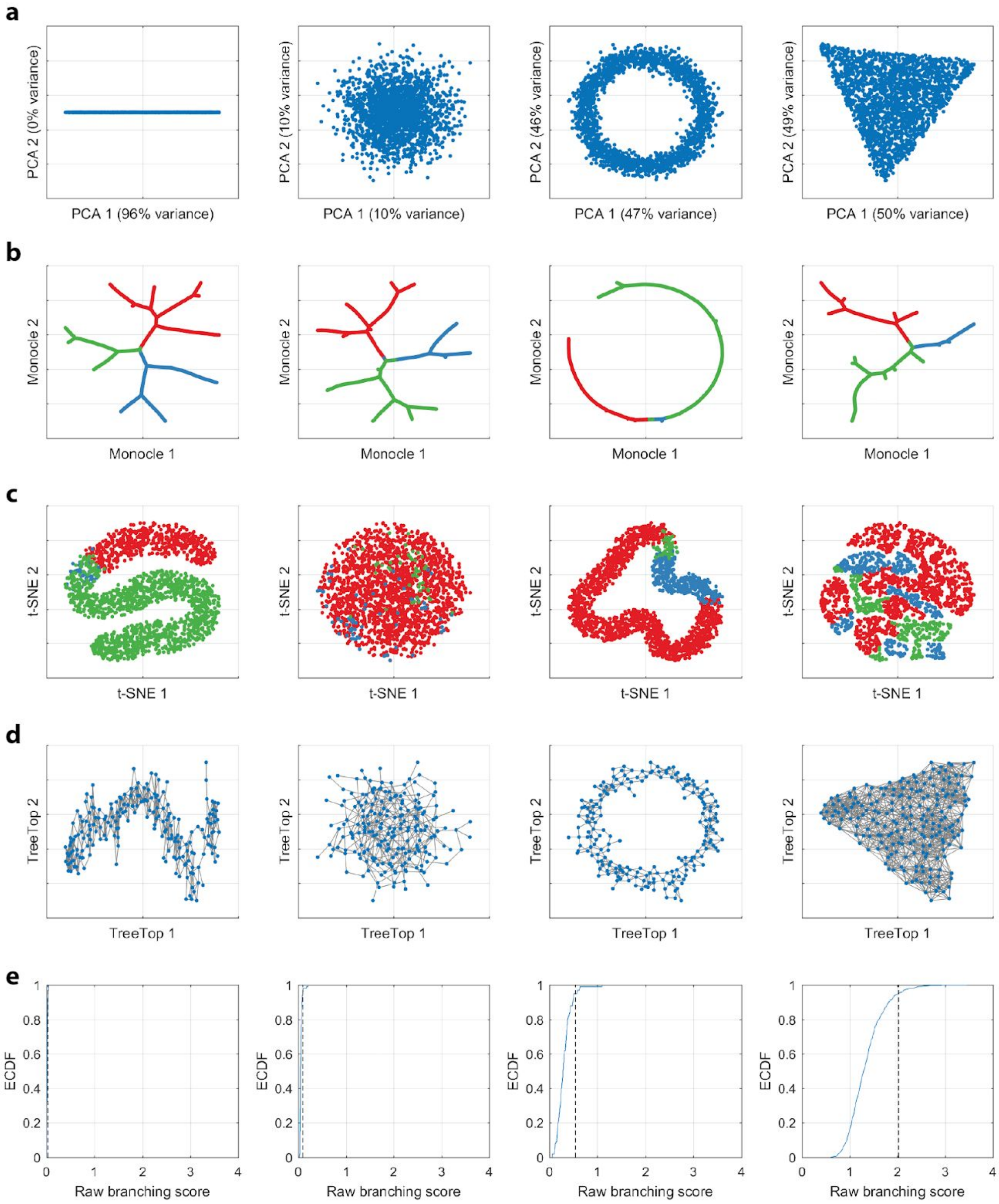


Each column corresponds to one simple synthetic non-branching dataset, from left to right: linear, Gaussian, circular, triangular. Each example dataset analysed here has 100,000

points, in 10 dimensions. **a** First two PCA components of each sample dataset (since the data is 10 dimensional, the variance explained by the first two components may not sum to 100%). Axis ranges scaled according to proportion of variance explained by component. **b** Monocle applied to sample of 2000 cells from each dataset. Colours represent branches identified at branch point with largest minimum branch size (this branch point manually selected). Layout from Monocle. **c** Wishbone applied to each dataset. Colours are branches identified by Wishbone (default parameters used, applied to whole dataset). Start cell for Wishbone selected manually to be extreme datapoint for each dataset: end of branch for linear, distant from mean in Gaussian, outside of circle, and corner for triangle. **d** TreeTop applied to each dataset, showing reference nodes connected by graph calculated from superposition of trees identified. **e** Distribution of maximum raw branching scores observed over 1000 samples of each synthetic dataset topology.

Appendix Figure S2: TreeTop applied to T cell thymic maturation data, with full markers

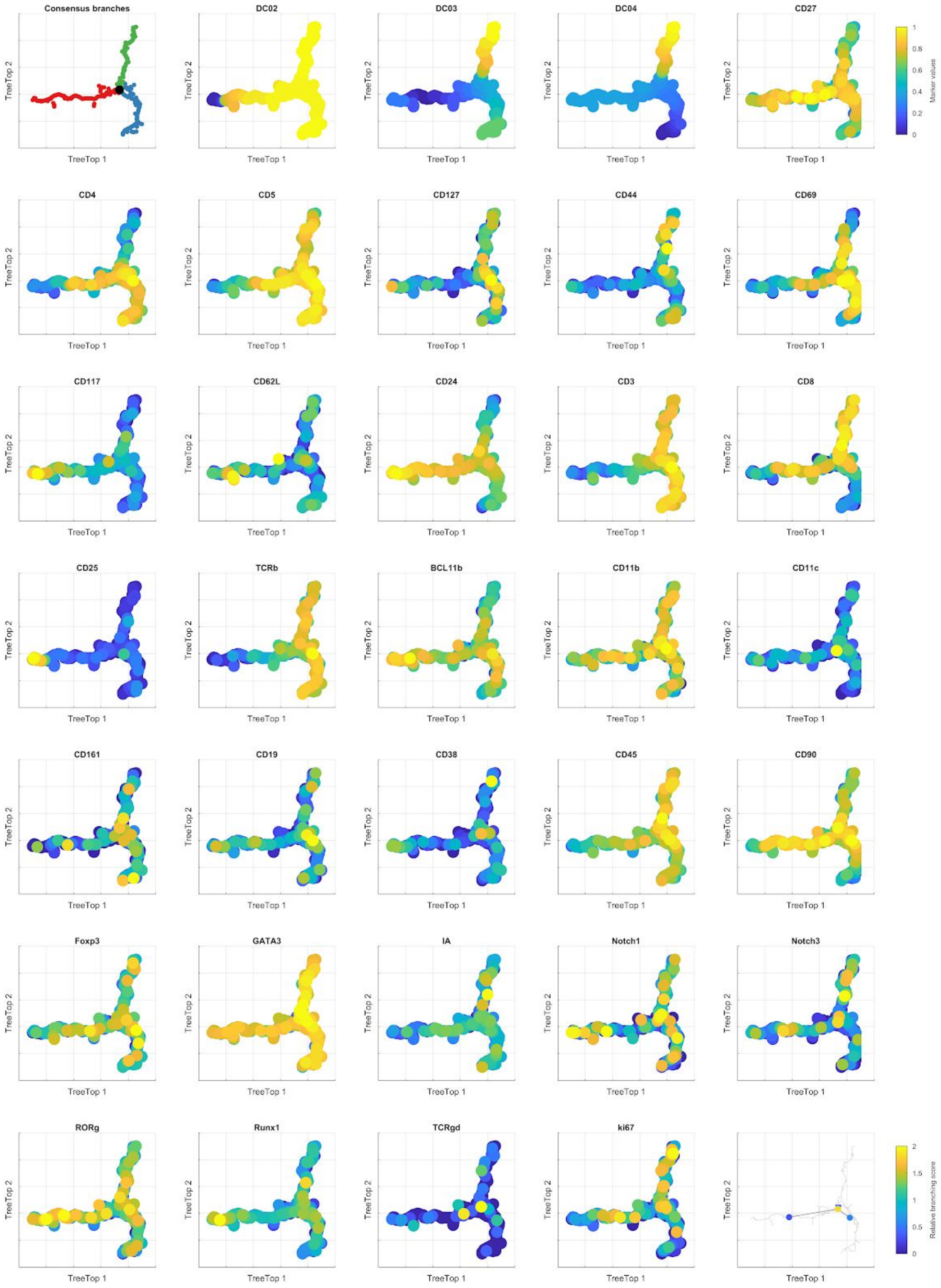

TreeTop applied to mass cytometry of maturing T cells sampled from the thymus, pre-processed via diffusion components (Setty *et al*, 2016). First plot shows branches identified by TreeTop, using TreeTop layout. Each point is a reference node, coloured by assigned branch; black point is branch point with highest relative branching score. Remaining plots (except last) show mean variable abundances at each reference node, using same layout. Values are arcsinhed protein abundance values, scaled to [0, 1]. Variables DC02, DC03, DC04 are diffusion components. TreeTop was applied to diffusion components; other variables are shown to aid interpretation. Diffusion components were calculated using all other variables as inputs (as done in (Setty *et al*, 2016)). Final plot shows results of recursive application of TreeTop: within each identified branch, the point with the highest relative branching score is identified, and an edge is drawn to the parent branch point.

## Appendix Figure S3: TreeTop applied to B cell maturation data, with full markers

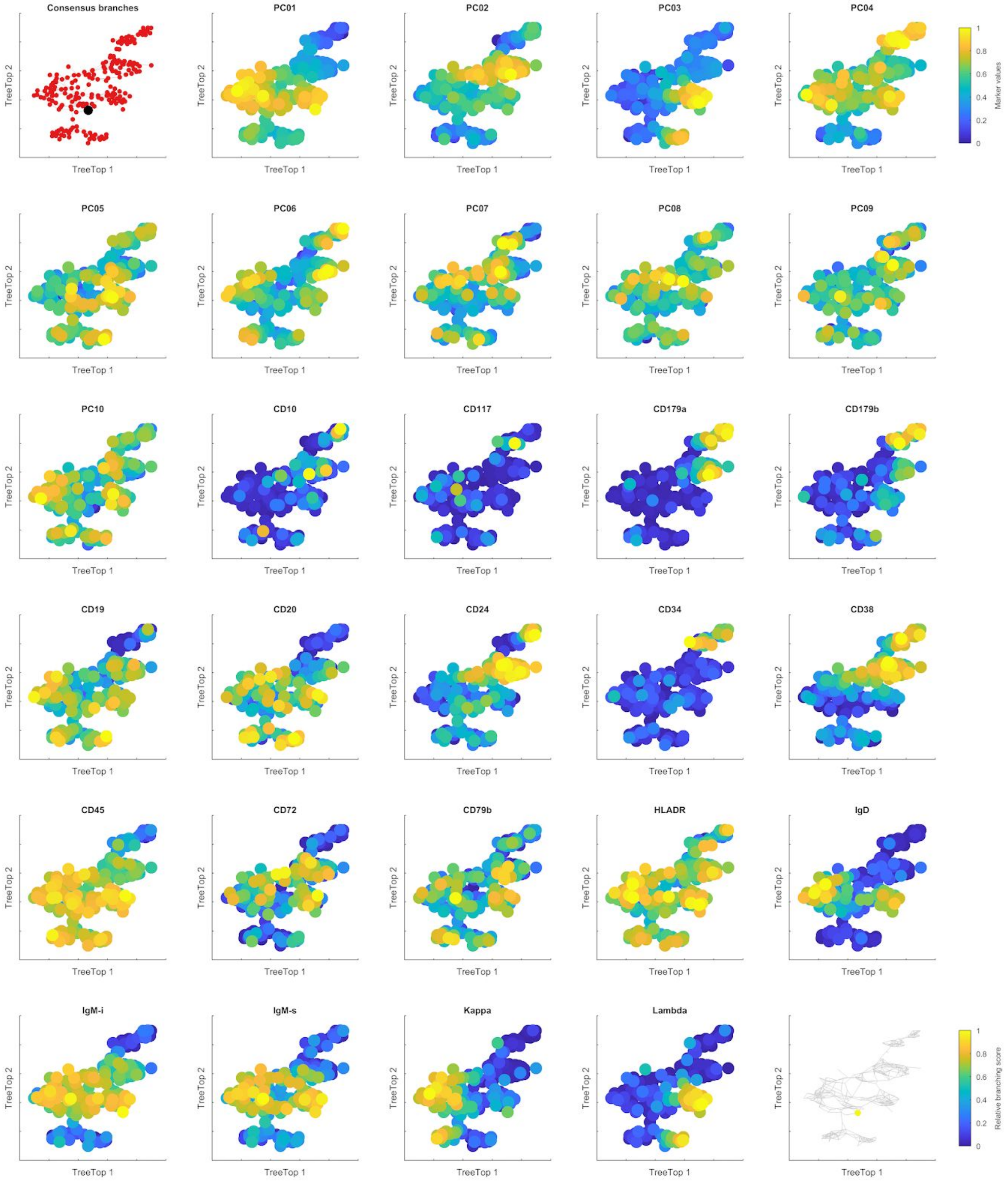


TreeTop applied to mass cytometry of differentiating B cells (Bendall *et al*, 2014). TreeTop applied to first 10 principal components of data; additional markers shown to aid interpretation. First plot shows branches identified by TreeTop, using TreeTop layout. Each point is a reference node, coloured by assigned branch; black point is branch point with highest relative branching score. Here, no branches were identified, so only one branch point is shown. Remaining plots (except last) show mean variable abundances at each

reference node, using same layout. Values are arcsinhed protein abundance values, scaled to [0, 1]. Final plot shows results of recursive application of TreeTop: within each branch, the point with the highest relative branching score is identified, and an edge is drawn to the parent branch point. Plots of markers show that the differentiating B cells follow several known changes in protein expression: loss of CD34 (a stem cell marker), transient expression of CD179, higher levels of CD19 and CD20 as the cells become more committed to the B cell lineage, and finally expression of either kappa or lambda light-chain antibody subunits. TreeTop does not identify a branch point, although the branch score is 0.97, which is just below the cutoff of 1 and could be interpreted as weak evidence in favour of branching. The visualization clearly shows the start of a separation into kappa and lambda light chain-committed cells, consistent with this interpretation.

Appendix Figure S4: Wishbone applied to T cell thymic maturation data, with full markers

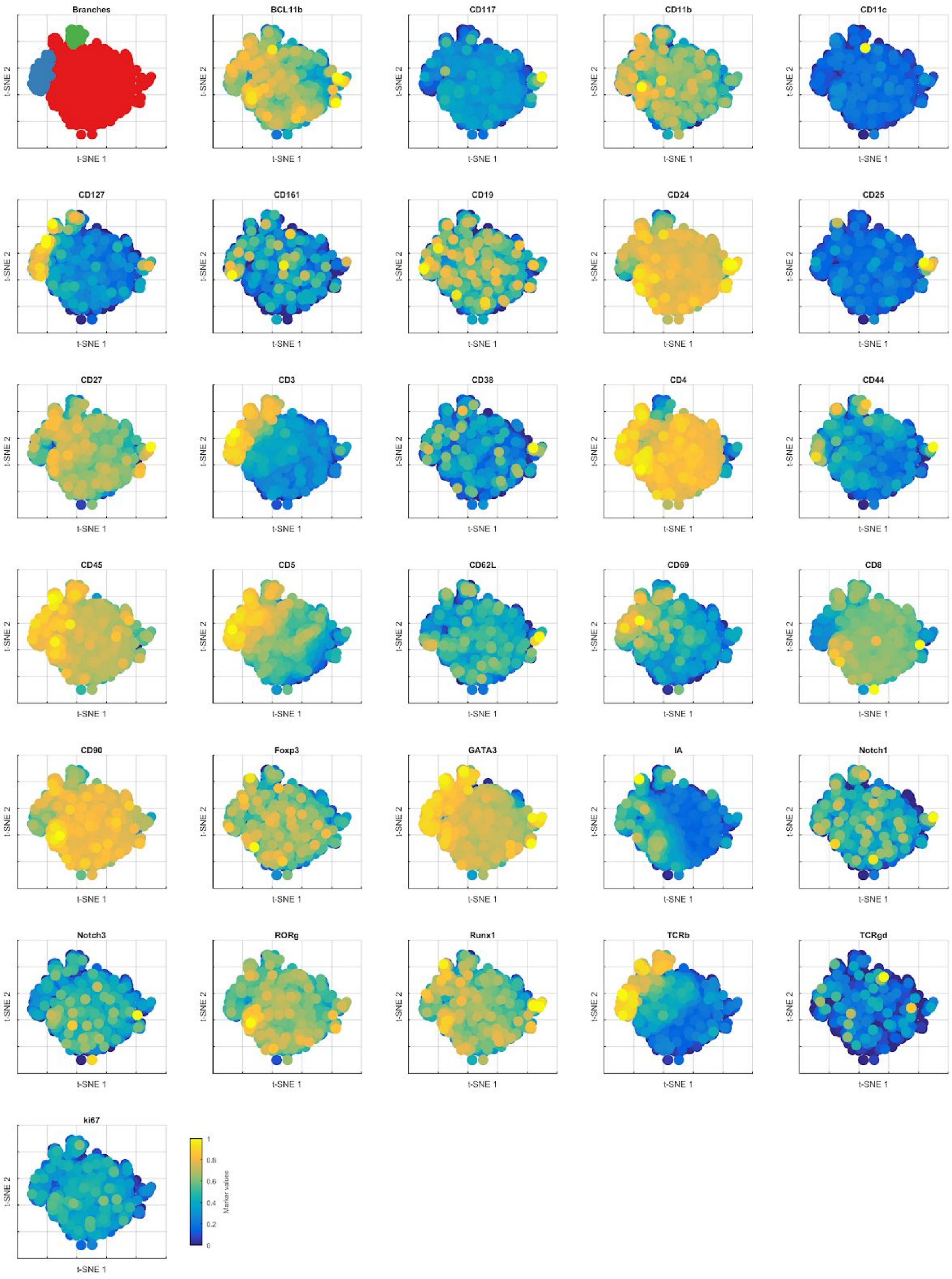

Wishbone applied to mass cytometry of maturing T cells sampled from the thymus (Setty *et al*, 2016). First plot shows branches identified by Wishbone, using t-SNE layout (Maaten & Hinton, 2008). Remaining plots show variable abundances at each cell, using same layout. Values are arcsinhed protein abundance values, scaled to [0, 1].

## Appendix Figure S5: Wishbone applied to B cell maturation data, with full markers

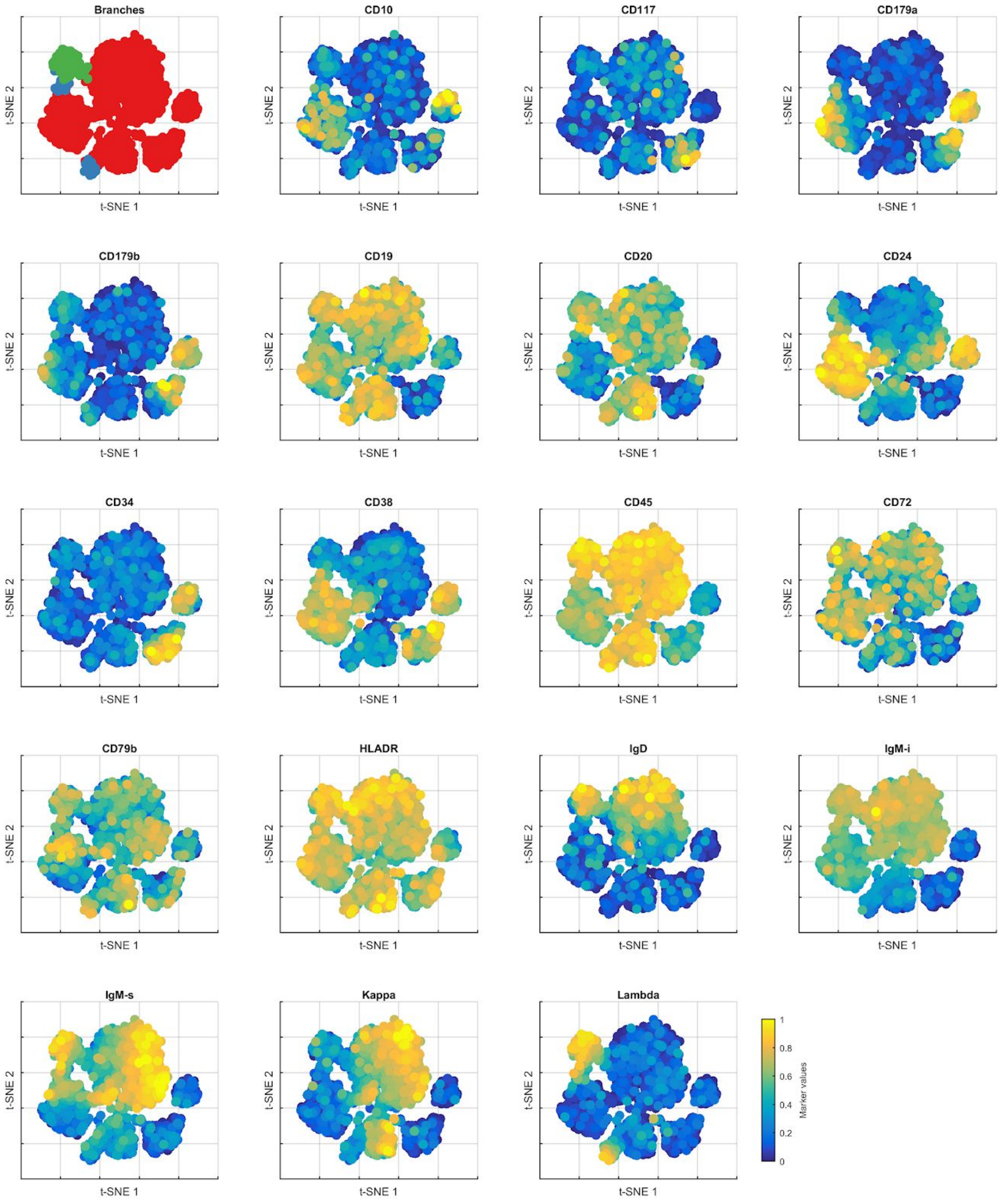


Wishbone applied to mass cytometry of differentiating B cells (Bendall *et al*, 2014). First plot shows branches identified by Wishbone, using t-SNE layout (Maaten & Hinton, 2008). Remaining plots show variable abundances at each cell, using same layout. Values are arcsinhed protein abundance values, scaled to [0, 1].

Appendix Figure S6: Monocle applied to T cell thymic maturation data, with full markers

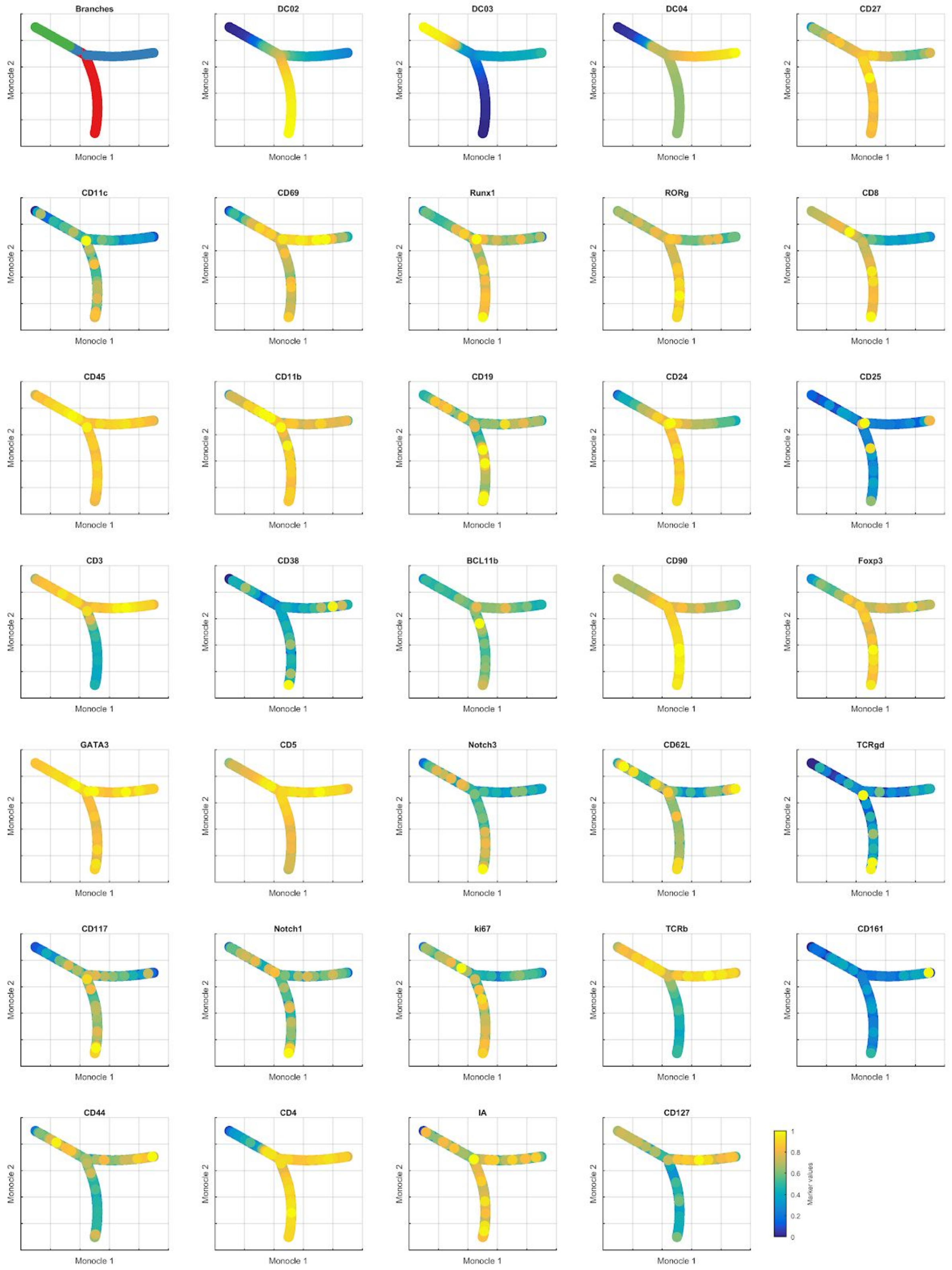

Monocle applied to mass cytometry of maturing T cells sampled from the thymus, pre-processed via diffusion components (Setty *et al*, 2016). First plot shows branches identified by Monocle, using Monocle layout; branch point selected manually. Remaining plots show variable abundances, using same layout. Values are arcsinhed protein abundance values, scaled to [0, 1]. Variables DC02, DC03, DC04 are diffusion components. Monocle was applied to diffusion components; other variables are shown to aid interpretation. Diffusion components were calculated using all other variables as inputs (as done in (Setty *et al*, 2016)).

## Appendix Figure S7: Monocle applied to B cell maturation data, with full markers

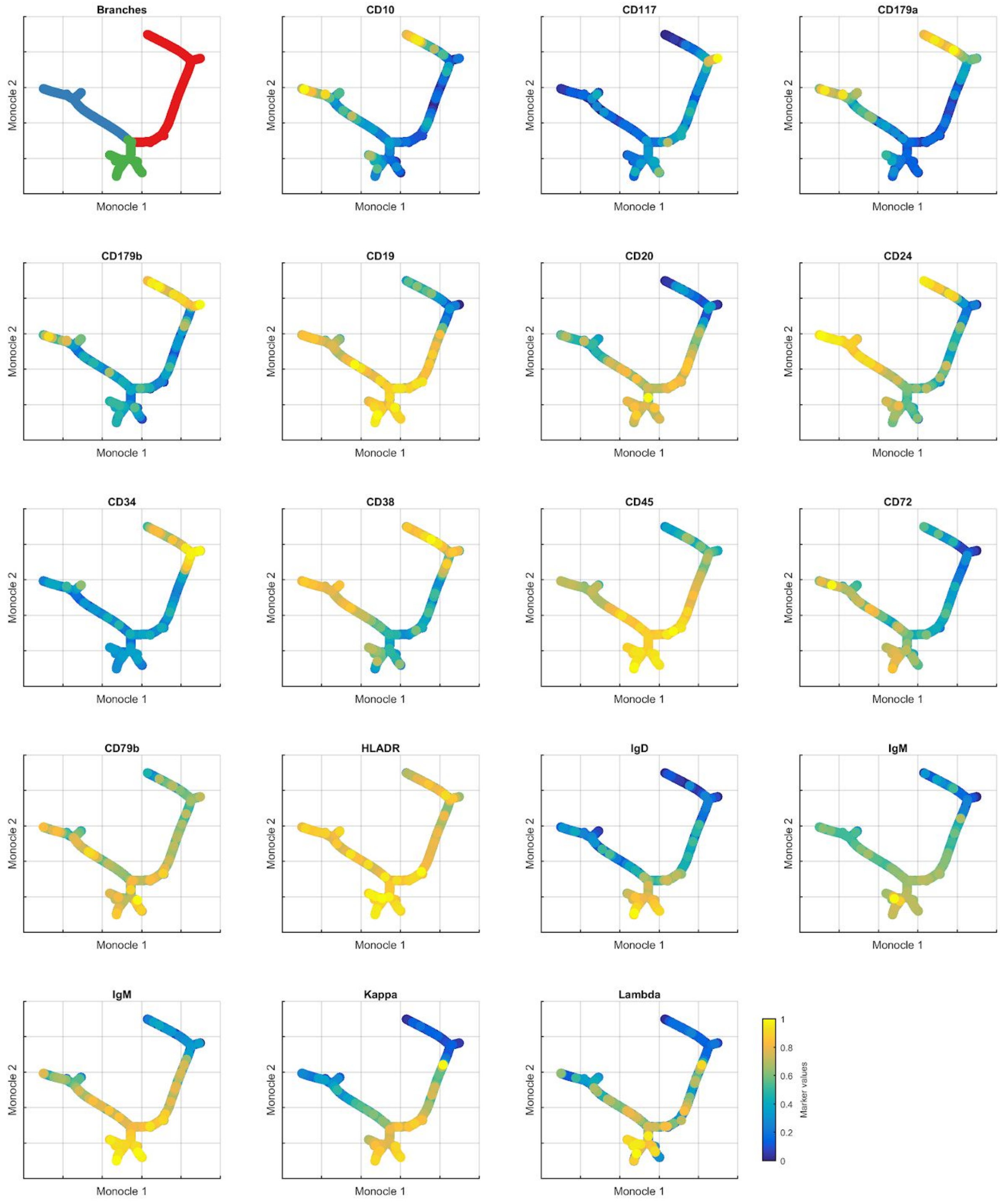


Monocle applied to mass cytometry of differentiating B cells (Bendall *et al*, 2014). First plot shows largest branches identified by Monocle, using Monocle layout; branch point selected manually. Remaining plots show mean variable abundances at each reference node, using same layout. Values are arcsinhed protein abundance values, scaled to [0, 1].

# Appendix Figure S8: TreeTop applied to healthy bone marrow data, with full markers

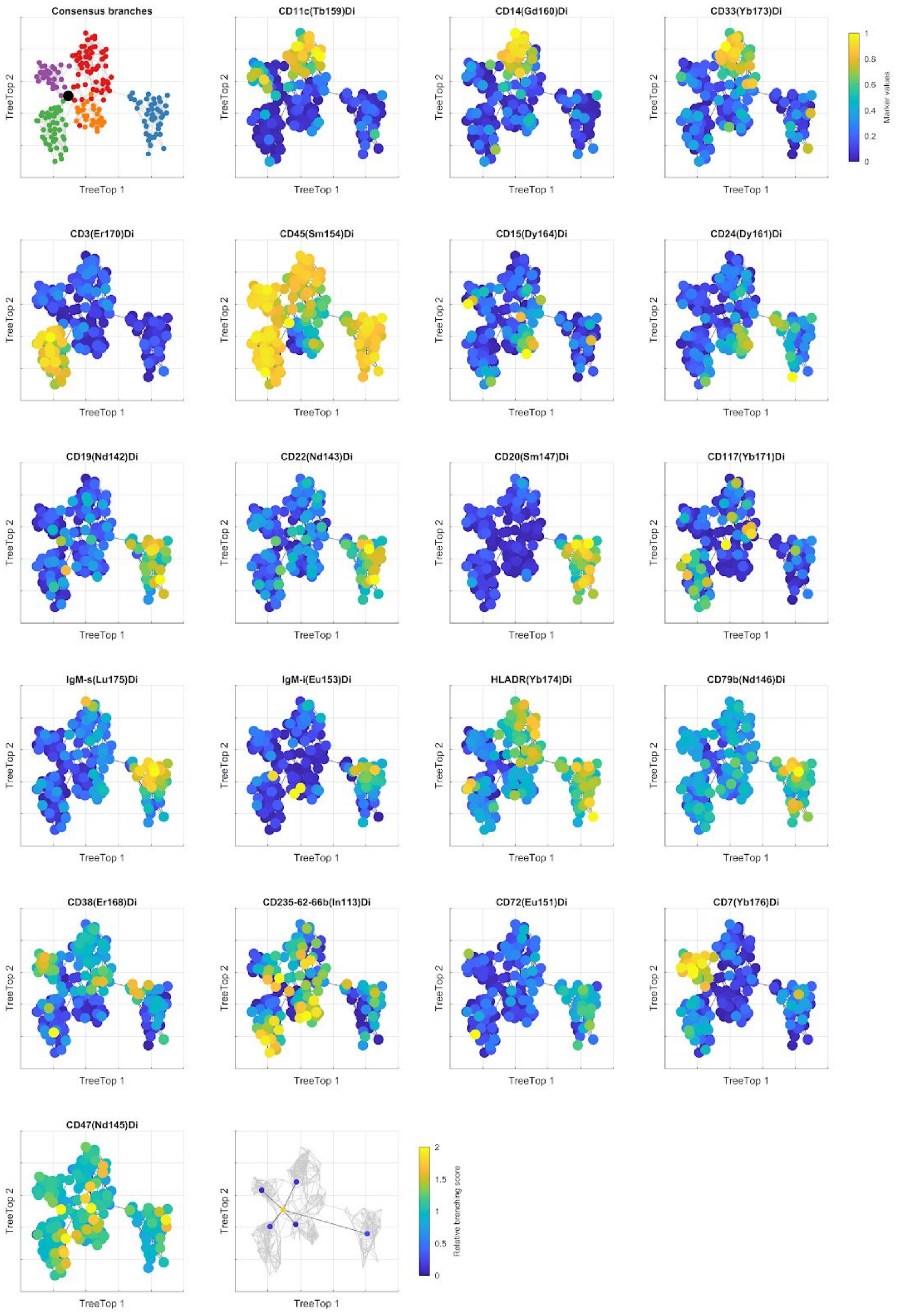

TreeTop applied to mass cytometry data sampled from healthy human bone marrow (Amir *et al*, 2013). First plot shows branches identified by TreeTop, using TreeTop layout. Each point is a reference node, coloured by assigned branch; black point is branch point with highest relative branching score. Remaining plots (except last) show mean variable abundances at each reference node, using same layout. Values are arcsinhed protein abundance values, scaled to [0, 1]. Final plot shows results of recursive application of TreeTop: within each branch, the point with the highest relative branching score is identified, and an edge is drawn to the parent branch point.

# Appendix Figure S9: Gating strategy for healthy bone marrow data

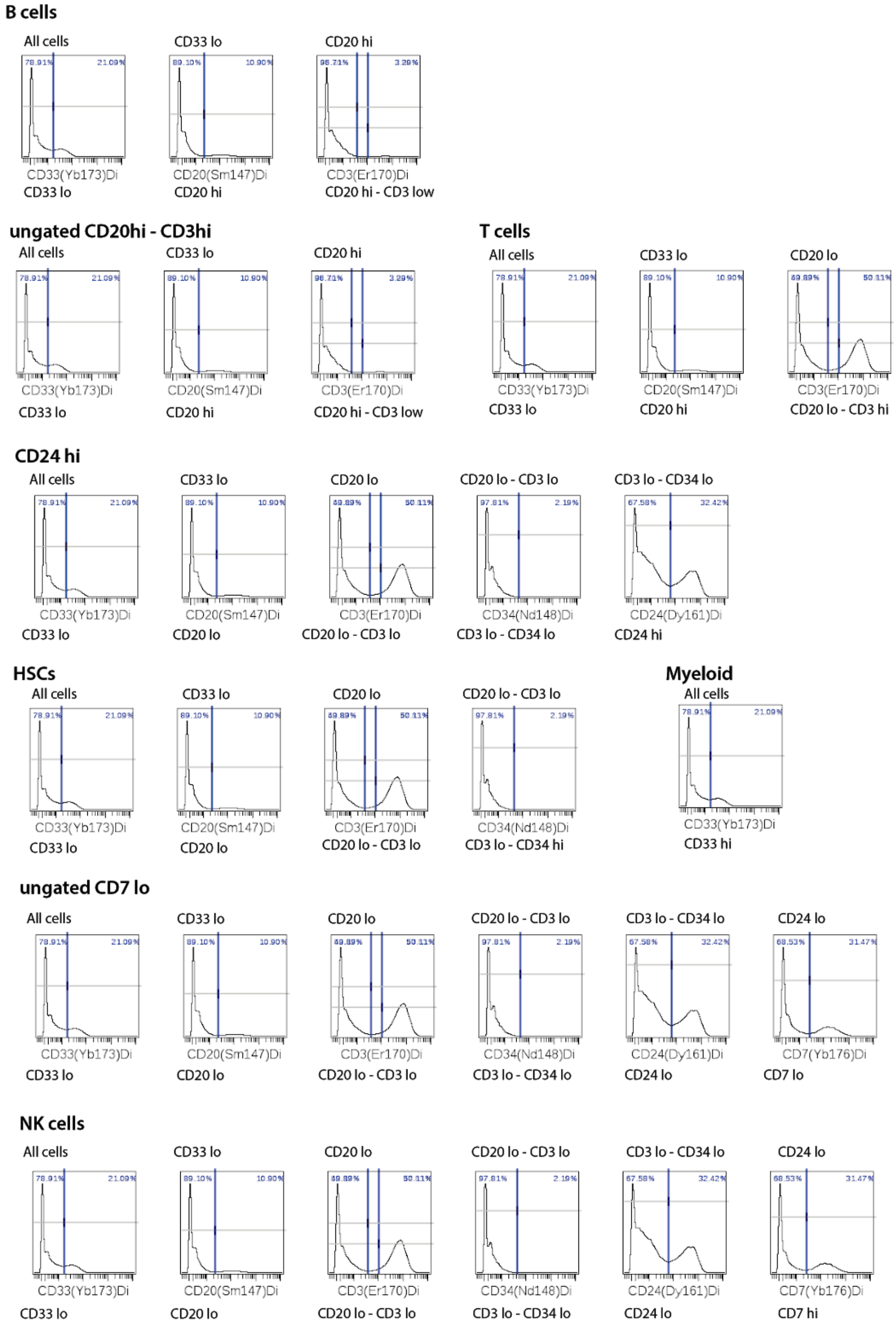


Gating strategy applied in Cytobank as follows: (1) CD33 high defined as Myeloid; (2) within CD33 low, CD20 high / CD3 low defined as B cells; (3) within CD20 low / CD33 low, CD3 high defined as T cells; (4) within CD3 low, CD34 high defined as HSCs; (5) within CD34 low, NK cells defined as CD24 low / CD7 high.

## Appendix Figure S10: TreeTop applied to synthetic branching data, with full markers

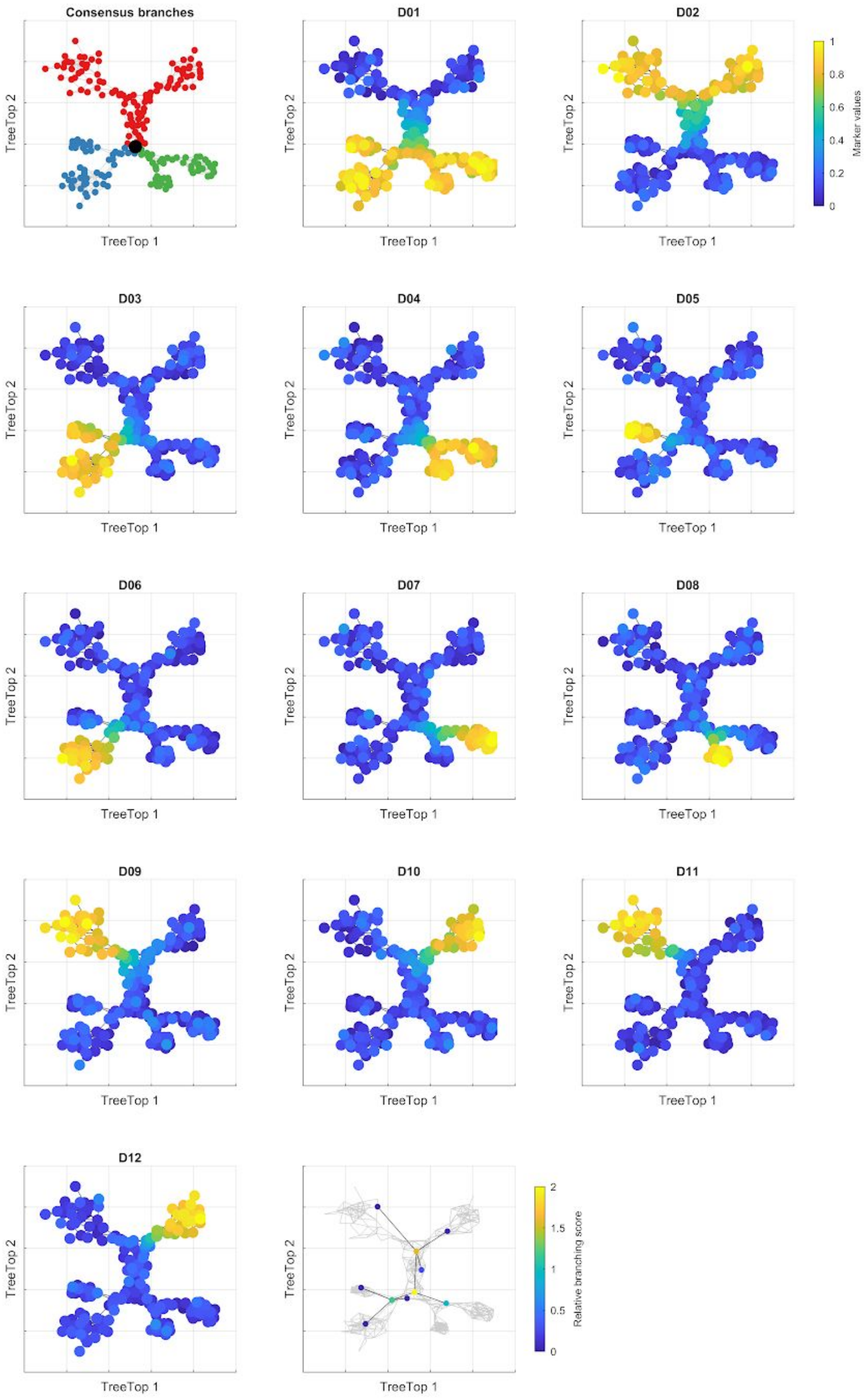


TreeTop applied to hierarchically branching synthetic data. First plot shows branches identified by TreeTop, using TreeTop layout. Each point is a reference node, coloured by assigned branch; black point is branch point with highest relative branching score. Remaining plots (except last) show mean variable abundances at each reference node,

using same layout. Values are arcsinhed simulated protein abundance values, scaled to [0, 1]. Final plot shows results of recursive application of TreeTop: within each branch, the point with the highest relative branching score is identified, and an edge is drawn to the parent branch point.

# Appendix Figure S11: Comparison of molecular species abundance distributions of synthetic branching data with mass cytometry data

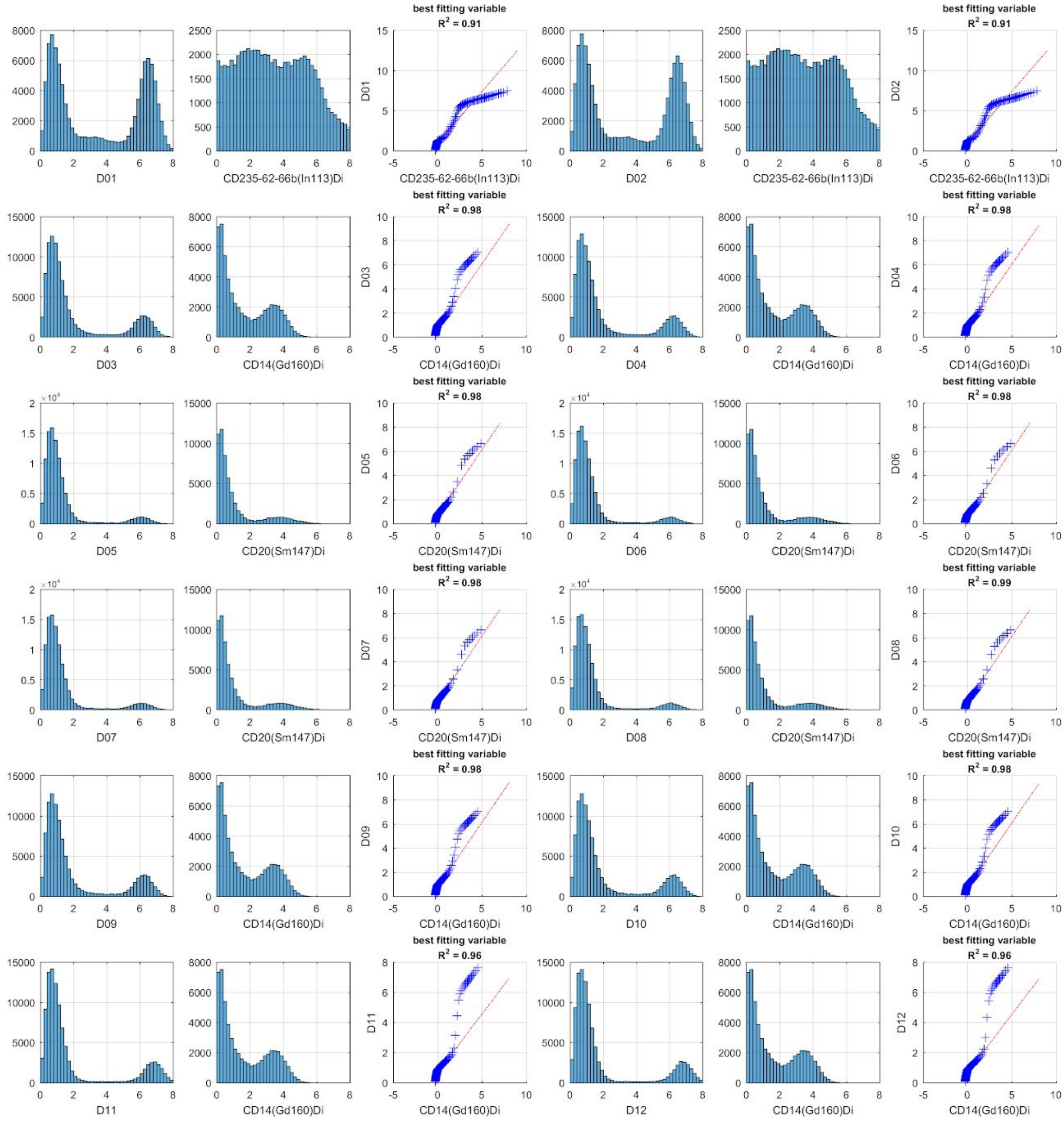


Plot shows marginal abundance distributions of each synthetic species (1st, 4th columns), after application of arcsinh with cofactor 5. Each species is matched to closest species distribution amongst the protein species recorded in the healthy bone marrow dataset (Amir *et al*, 2013). The matching is achieved by calculating the 1%, 2% through to 99% quantiles of the data distribution of the synthetic species, and of all the real protein species. A regression is then fit to explain the synthetic data quantiles from the quantiles of each real protein species, and the $R^2$ value of this fit calculated. Columns 2 and 5 show the marginal data distribution of the real protein species with the highest $R^2$ value for that synthetic species. Columns 3 and 5 show scatter plots of the quantiles of the synthetic species against the best matching protein species, with the accompanying $R^2$ value.

Appendix Figure S12: Distribution of annotated celltypes in TreeTop applied to healthy human bone marrow single cell RNA-seq data from Paul *et al*

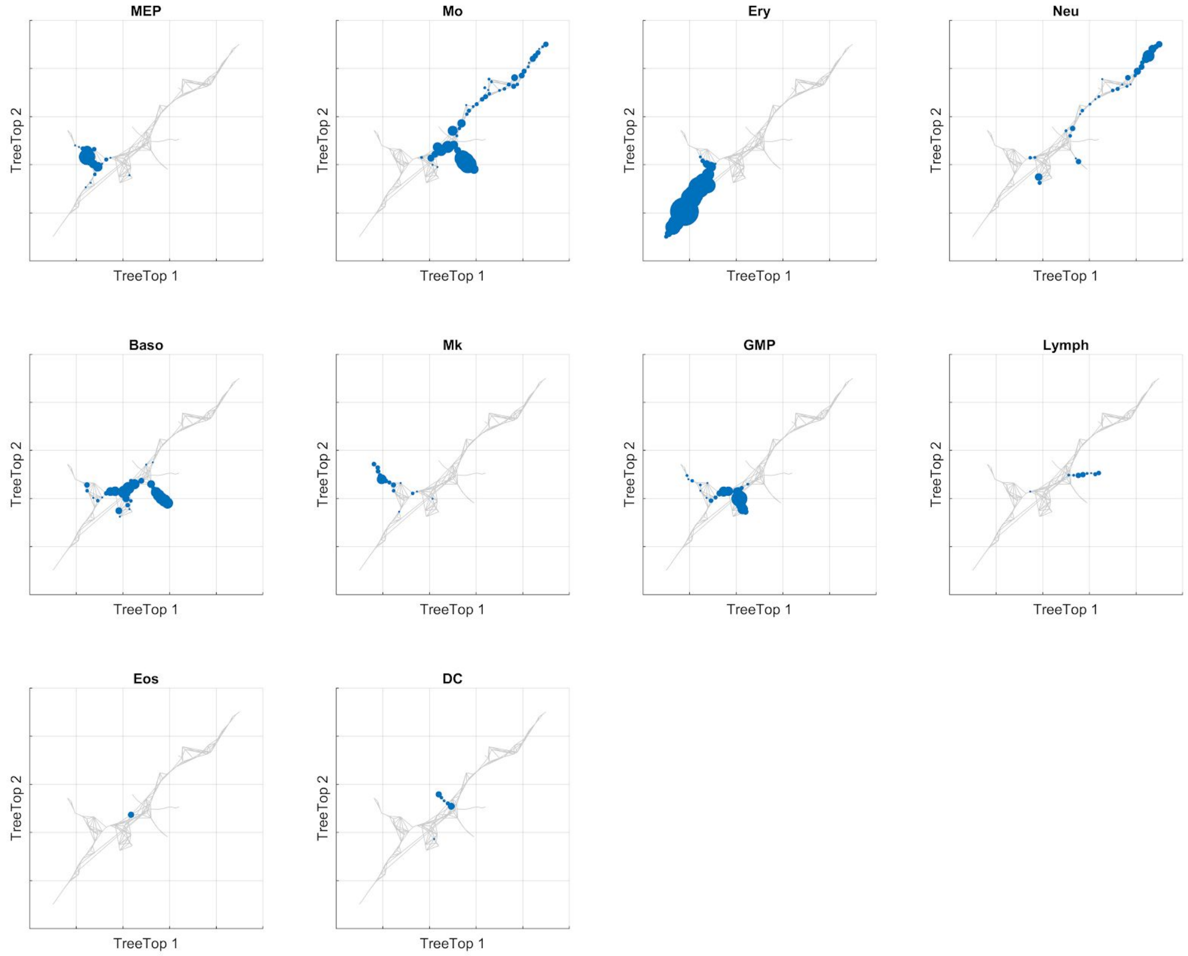


TreeTop applied to single cell RNA-seq data derived from 2730 haematopoietic stem cells taken from healthy human bone marrow (Paul *et al*, 2015). TreeTop applied to diffusion map processed data. Overlay of TreeTop output with cell labels from paper: MEP=Megakaryocyte-erythroid progenitor, Mo=Monocyte, Ery=Erythroid, Neu=Neutrophil, Baso=Basophil, Mk=Megakaryocyte, GMP=Granulocyte-macrophage progenitor, Lymph=Lymphoid, Eos=Eosinophil, DC=Dendritic Cell. Labels not used as input to TreeTop. Area of point proportional to number of cells of that label at that node.

Appendix Figure S13: Distribution of annotated celltypes in TreeTop applied to healthy human bone marrow single cell RNA-seq data from Velten *et al*

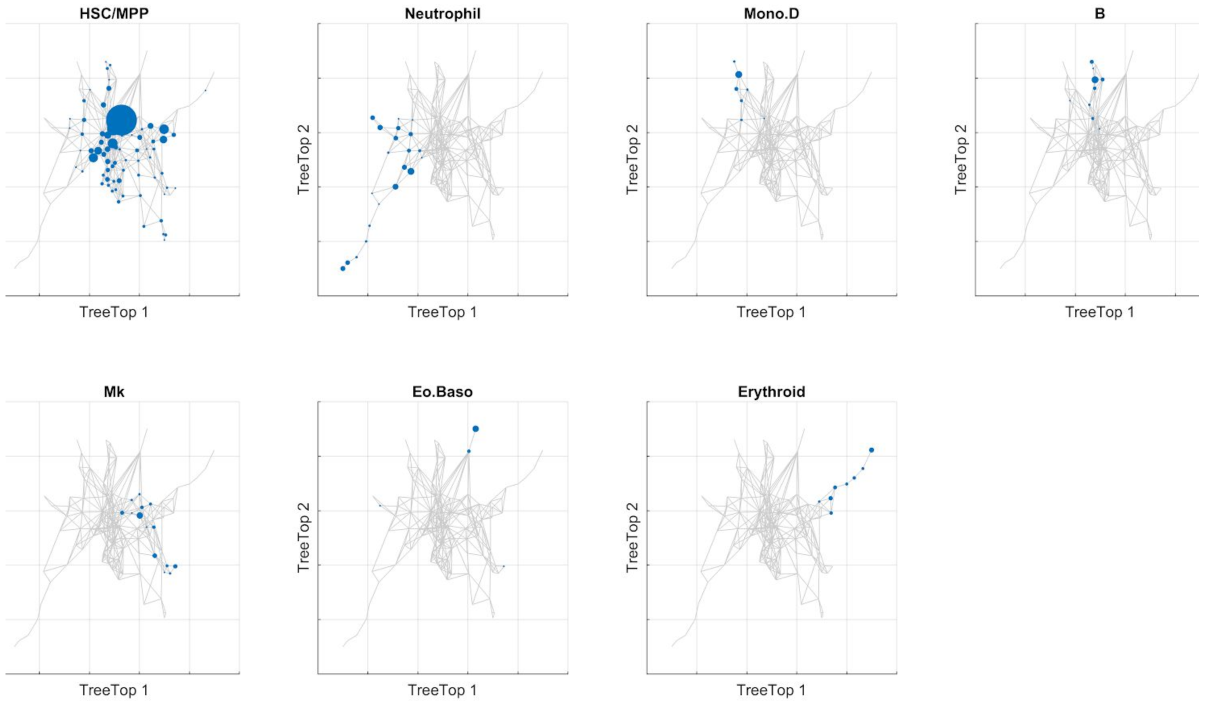


TreeTop applied to single cell RNA-seq data derived from 1034 haematopoietic stem and pluripotent cells taken from healthy human bone marrow (Velten *et al*, 2017). Data from donor 1 used, preprocessed (variance stabilizing transform) as in original paper. TreeTop applied to diffusion map processed data. Overlay of TreeTop output with cell labels from paper: Mono-D=Monocyte / Dendritic Cell, B=B cell, Mk=Megakaryocyte, Eo.Baso = Eosinophil / Basophil. Labels not used as input to TreeTop. Area of point proportional to number of cells of that label at that node.

# Appendix Figure S14: Robustness of TreeTop to number of reference nodes

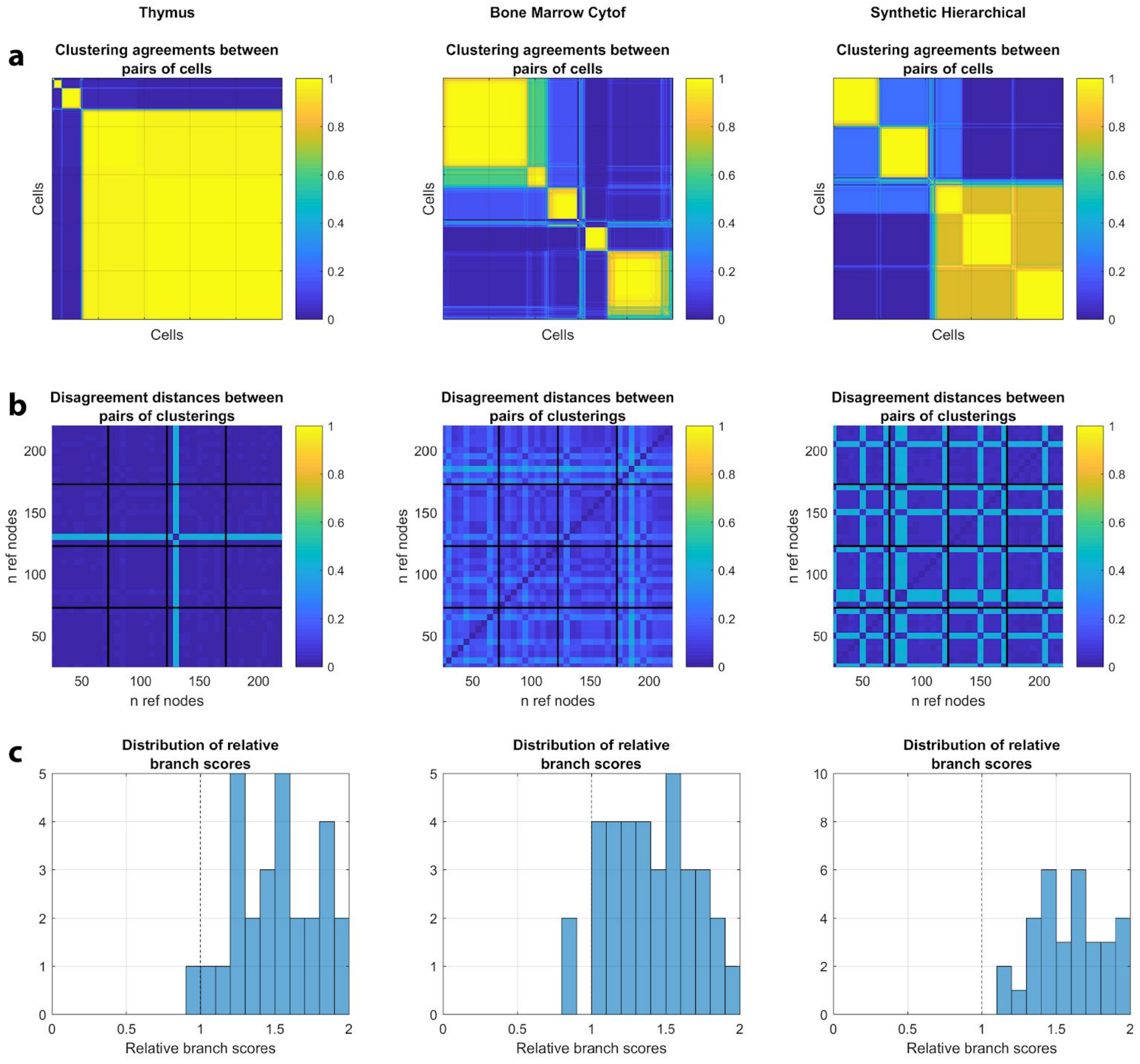


TreeTop run on same data used for results shown in **Figures 1e-g** (thymus)**, 2a** (healthy human bone marrow) and **2e** (synthetic hierarchical branching), with 50, 100, 150 and 200 reference nodes, for 10 different seeds each (40 runs in total per dataset). **a** Consensus clustering comparison between pairs of cells. 5000 cells selected at random, colour shows proportion of the 40 runs in which these cells were placed in the same branch. For a pair of cells *(i, j)*, matrix shows values close to 0 when *i* and *j* were rarely put in the same branch across the 40 runs, and values close to 1 when they were often put in the same branch. The matrices are block diagonal, corresponding to consistently identified branches. **b** Comparison of disagreement between identified branches. For each run, a binary matrix is calculated where *(i,j)* = 0 if cells *i* and *j* were in different branches, and *(i,j)* = 1 if in the same branch. Disagreement shown here is the Hamming distance between the binary matrices for each pair of these runs. Black lines separate the different values of n ref nodes used; within each such section the results of the 10 different random seeds are shown. **c** Distributions of maximum observed relative branching scores across all 40 runs.

## Appendix Figure S15: Distributions of raw branching scores on permuted data

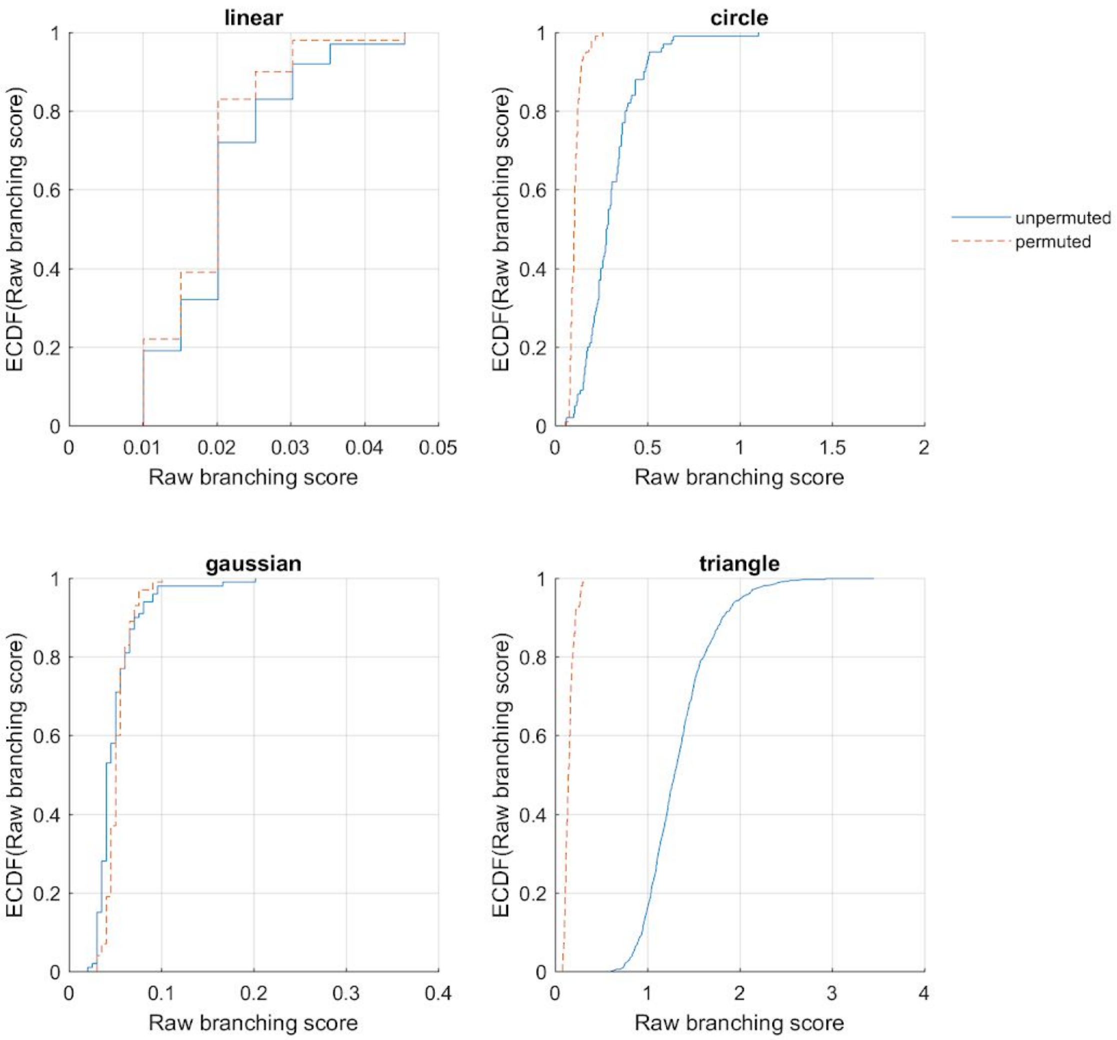


Blue, solid lines show maximum raw branching score for each dataset generated for synthetic non-branching distributions with simple topologies (distributions shown here are identical to those in **Appendix Figure S1e**). Red, dashed lines show distributions of 100 permutations of one of these datasets. Datasets were permuted by randomly reordering the set of values for each input dimension. In all cases except linear, the permuted data has much lower raw branching scores. In the linear example, any branches identified were so small and occurred over so few dendrogram thresholds that the distribution becomes discrete.

## Appendix Figure S16: Effect of dimensionality and topology of synthetic data on raw branching scores

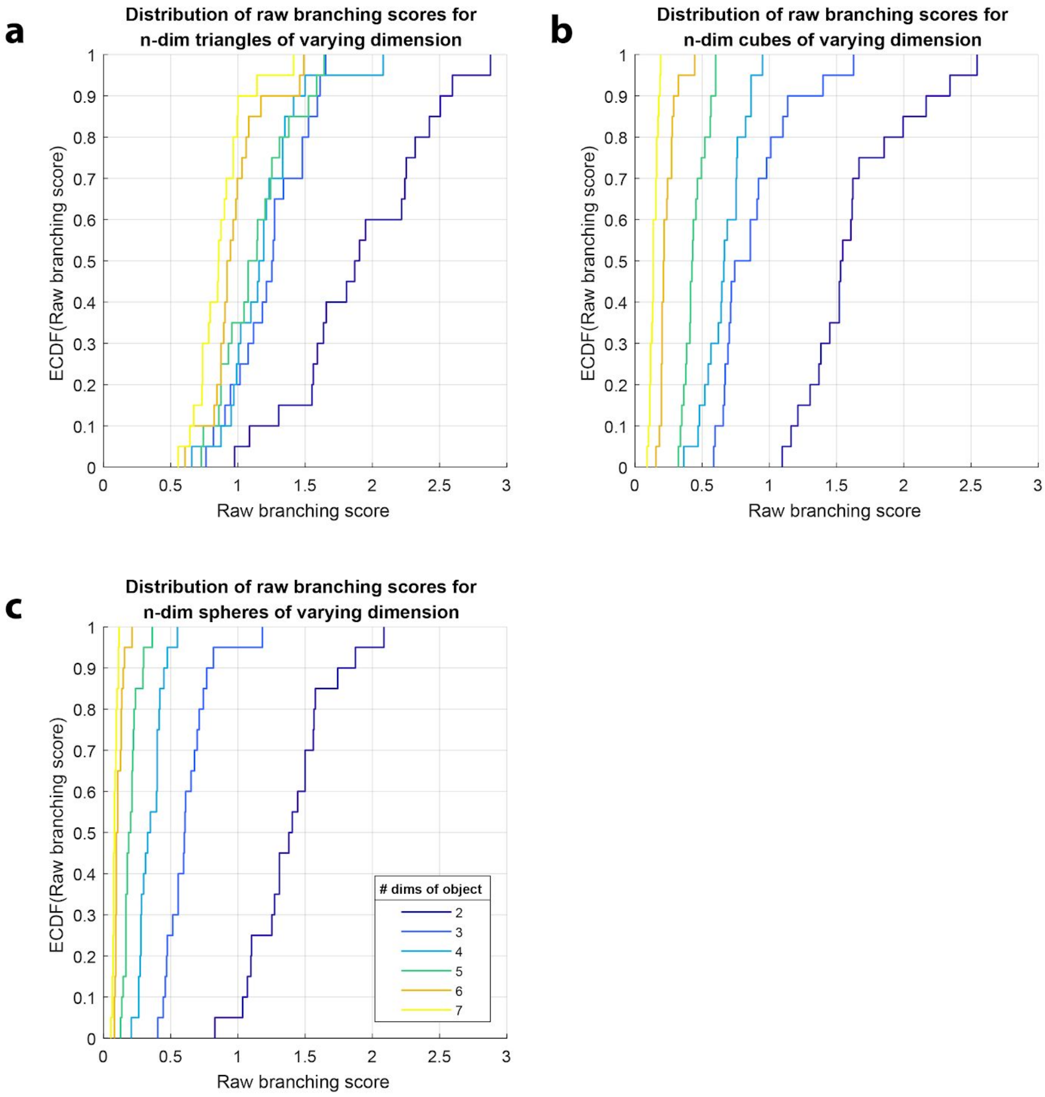


Empirical cumulative distribution functions (ECDFs) of maximum raw branching scores observed in simple synthetic data with increasing dimensionality. Each line shows the distribution of maximum raw branching scores observed in runs of TreeTop applied to 20 randomly generated datasets with the specified dimensionality and class of geometric object. The classes of geometric object are **a** simplices (generalizations of triangles to higher dimensions), **b** n-dimensional cubes and **c** n-dimensional spheres. Each dataset comprises 100,000 points uniformly sampled from the interior of the object with the defined number of dimensions, embedded within a larger (30-dimensional) space, with no measurement noise. All TreeTop runs use 200 reference nodes.

# Appendix Figure S17: Effect of input parameters on reference score distributions

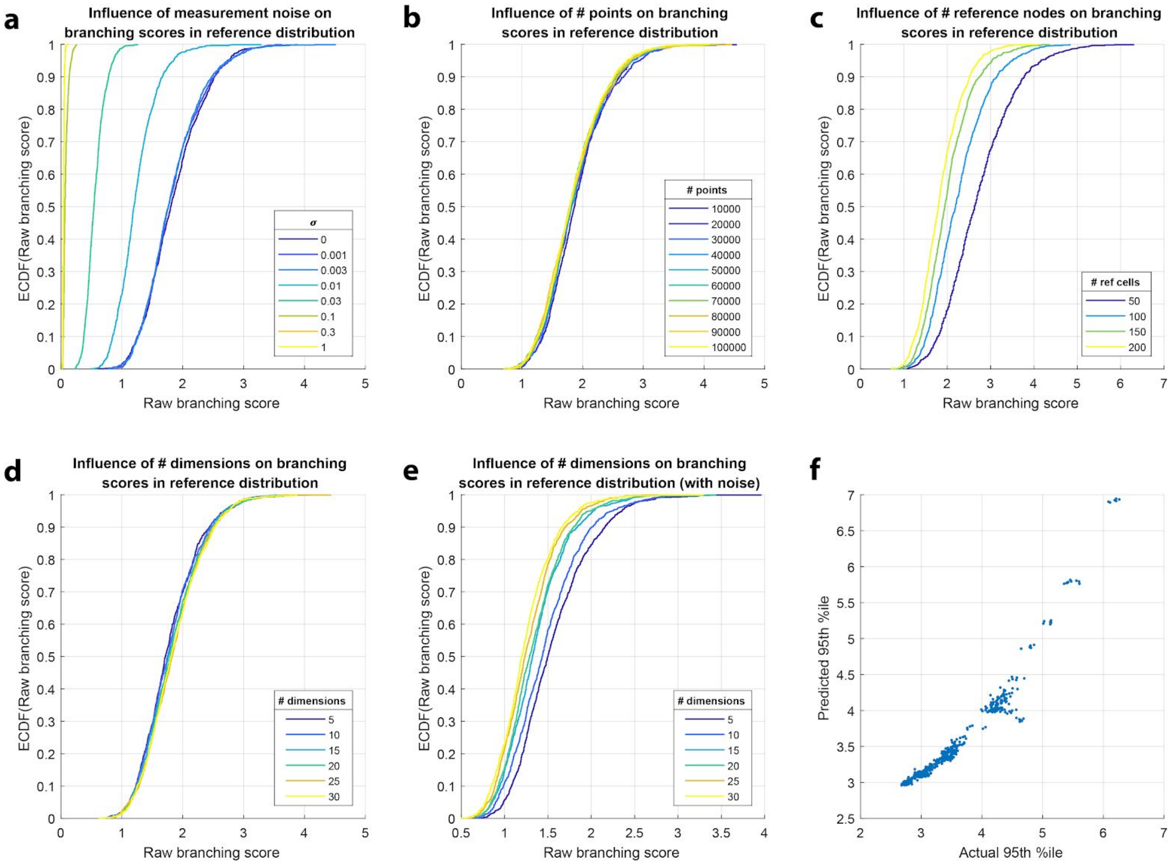


**a** Empirical cumulative score distribution functions (ECDFs) of maximum raw branching scores observed in reference score distributions with varying levels of simulated Gaussian measurement noise. All runs are triangular data, with 100,000 data points, 30 dimensions, 200 reference nodes. **b** ECDFs of maximum raw branching scores observed in reference score distributions with varying numbers of datapoints. All runs are triangular data, with 0 measurement noise, 20 dimensions, 200 reference nodes. **c** ECDFs of maximum raw branching scores observed in reference score distributions with varying numbers of reference nodes. All runs are triangular data, with 0 measurement noise, 20 dimensions, 100,000 data points. **d** ECDFs of maximum raw branching scores observed in reference score distributions with varying numbers of dimensions. All runs are triangular data, with 0 measurement noise, 100,000 data points, 200 reference nodes. **e** ECDFs of maximum raw branching scores observed in reference score distributions with varying numbers of datapoints, in the presence of Gaussian measurement noise with σ = 0.01. All runs are triangular data, 100,000 data points, 200 reference nodes. **f** 95th percentiles of maximum raw branching scores observed in reference score distributions were predicted, using number of dimensions, 1/number of datapoints, and 1/number of reference nodes as inputs into MATLAB's *fitlm* linear model function. Plot shows actual 95th percentile values vs predicted. Standard output from *fitlm* function was as follows:

```
Linear regression model:
```

```
    q95 ~ 1 + n_dims + inv_points + inv_ref_cells
Estimated Coefficients:
                     Estimate       SE        tStat       pValue
                     ________    _________    ______    ___________

    (Intercept)        2.6142     0.025611    102.07    1.5066e-303
    n_dims           0.002121    0.0011284    1.8797       0.060818
    inv_points         956.17       31.627    30.233    1.8042e-108
    inv_ref_cells      63.587       1.3845    45.927    6.4274e-168
Number of observations: 434, Error degrees of freedom: 430
Root Mean Squared Error: 0.199
R-squared: 0.924,  Adjusted R-Squared 0.923
F-statistic vs. constant model: 1.74e+03, p-value = 4.11e-240
```

# Appendix Tables

## Appendix Table S1: Details of synthetic datasets

To generate exploratory datasets for testing possible reference score distributions, we used the parameters in the following table.

The absolute location within the defined space does not affect TreeTop analysis, which is based on relative distances between points. Each sample of the datasets described in **Appendix Figure S1** was generated from an n-dimensional space, where n was uniformly sampled from [6, 30].

| Dataset | Dataset description | Dimensionality | Number of points |
|---|---|---|---|
| Gaussian | n-dimensional standard normal distribution | Sampled uniformly from [6, 30] | 100,000 |
| Linear | First dimension is sampled from a uniform distribution, all other dimensions have value 0. n-dim standard normal noise added. Uniformly random n-dimensional rotation. | Sampled uniformly from [6, 30] | 100,000 |
| Circular | First two dimensions sampled uniformly from a circle of unit radius, all other dimensions have value 0. n-dim standard normal noise added. Uniformly random n-dimensional rotation. | Sampled uniformly from [6, 30] | 100,000 |
| Triangular | First two dimensions sampled uniformly from interior of an equilateral triangle of unit side length, all other dimensions have value 0. n-dim standard normal noise added. Uniformly random n-dimensional rotation. | Sampled uniformly from [6, 30] | 100,000 |

## Appendix Table S2: Generation of hierarchically branching synthetic data

| Parameter | Symbol | Value |
|---|---|---|
| Basal synthesis rate | $\alpha_0$ | 25 |
| synthesis rate | $\alpha$ | 20000 |

| | | |
|---|---|---|
| decay rate | λ | 0.25 |
| dissociation constant (activation) | $\kappa_+$ | 20000 |
| Hill coefficient (activation) | $h_+$ | 2 |
| dissociation constant (inhibition) | $\kappa_-$ | 100 |
| Hill coefficient (inhibition) | $h_-$ | 1.5 |

## Appendix Table S3: TreeTop parameters

All runs sampled 1000 trees. TreeTop used either input variables as specified in the relevant original paper, or in the case of synthetic data, all variables.

Default values for TreeTop are:

- # reference nodes = 200
- # trees = 1000
- Density outlier threshold = 1%
- Density high threshold = 50%
- Distance used = L1
- Arcsinh cofactor = 5

Increasing the number of trees used improves the ability of TreeTop to identify branch points, however we have found 1000 trees to be sufficient.

| **Dataset** | **# input points (N)** | **# input variables (D)** | **# reference nodes** | **Density σ** | **Density outlier threshold** | **Density high threshold** | **Distance used** | **Pre-processed with diffusion maps?** |
|---|---|---|---|---|---|---|---|---|
| T cell thymic maturation (Setty *et al*, 2016) | 220,076 | 30 | 200 | 1e-4 | 0.01 | 0.2 | L1 | Yes |
| Healthy bone marrow (Amir *et al*, 2013) | 103,861 | 20 | 200 | 2 | 0.01 | 0.5 | L1 | No |
| Hierarchically branching synthetic data | 100,000 | 12 | 200 | 1 | 0.01 | 0.5 | L1 | No |
| Linear | 100,000 | 10 | 200 | 0.05 | 0.01 | 0.5 | L1 | No |
| Circle | 100,000 | 10 | 200 | 1 | 0.01 | 0.5 | L1 | No |
| Gaussian | 100,000 | 10 | 200 | 0.05 | 0.01 | 0.5 | L1 | No |

| | | | | | | | | |
|---|---|---|---|---|---|---|---|---|
| Triangular | 100,000 | 10 | 200 | 0.05 | 0.01 | 0.5 | L1 | No |
| B cell maturation (Bendall *et al*, 2014) | 19,291 | 10 | 200 | 0.05 | 0.01 | 0.2 | L1 | No |
| Paul *et al.* healthy bone marrow sc RNA-seq (Paul *et al*, 2015) | 2,730 | 1000 | 50 | 0.01 | 0 | 0.2 | L1 | Yes |
| Velten *et al.* healthy bone marrow sc RNA-seq (Velten *et al*, 2017) | 1,034 | 1000 | 100 | 1 | 0 | 0.1 | L1 | Yes |

## Appendix Table S4: Wishbone parameters

| **Dataset** | **Diffusion components used** | **Start cell specification** | **kNN for diffusion maps** |
|---|---|---|---|
| Healthy bone marrow(Amir *et al*, 2013) | 1,2,3,4,5 | CD3_Er170_Di > 3, CD14_Gd160_Di < 1 | 60 (default) |
| Linear | 1,2,3 | D01 > 0.5, D02 < 1 | 60 (default) |
| Gaussian | 1,2,3 | D01 > -3, D02 > 4 | 60 (default) |
| Circle | 1,2,3,4 | D01 > 0.9, D02 > 0.9 | 60 (default) |
| Triangle | 1,2,3,4,5 | D04 > 0.1, D05 > 0.05 | 60 (default) |
| T cell thymic maturation(Setty *et al*, 2016) | 1,2,3 | CD3 > 2, CD25 > 4 | 60 (default) |
| B cell maturation(Bendall *et al*, 2014) | 1,2,3,4,5 | CD34 > 2, CD117 > 0.5 | 60 (default) |

Default values were used for all other parameters.

## Appendix Table S5: Timings for comparison methods

| **Method** | **Number of cells in input** | **Mean** | **Standard deviation** |
|---|---:|---:|---:|
| TreeTop - one core | 100,000 | 345s | ±36s |
| TreeTop - four cores | 100,000 | 220s | ±28s |

| | | | |
|---|---|---|---|
| TreeTop - recursive | 100,000 | 655s | ±77s |
| Wishbone | 100,000 | 2060s | ±66s |
| Monocle 2 | 2,000 | 378s | ±40s |

Mean and standard deviation calculated over 10 runs with different seeds, on an Intel Core i7-6700 3.4GHz CPU with 16 GB RAM.
